# Correlations of serum myostatin and irisin with sarcopenia and osteoporosis in rheumatoid arthritis patients: a cross-sectional study

**DOI:** 10.1038/s41598-025-07378-8

**Published:** 2025-07-02

**Authors:** Wan-jun Li, Ruo-yan-ran Yin, Xiao-xuan Xia, Qian-xi Xing, Sheng-qian Xu

**Affiliations:** 1 Department of Endocrine Laboratory, The First Affiliated Hospital of Anhui Medical University, Hefei, China; 2 Department of Rheumatology and Immunology, The First Affiliated Hospital of Anhui Medical University, Hefei, China

**Keywords:** Rheumatoid arthritis, Sarcopenia, Osteoporotic fractures, Serum myostatin levels, Serum irisin levels

## Abstract

Myostatin and irisin, the key regulators of muscle metabolism, are myokines secreted by skeletal muscles that significantly influence osteoporosis (OP) and sarcopenia. This study investigated the associations of serum myostatin and irisin levels with sarcopenia and OP prevalence in 182 rheumatoid arthritis (RA) versus 142 healthy controls. RA patients exhibited significantly elevated serum myostatin (*P* = 0.001) and reduced irisin levels (*P* = 0.009), with markedly higher rates of myostatin/irisin dysregulation compared to controls (both *P* < 0.0001). Subgroup analyses revealed that RA patients with sarcopenia had higher myostatin (*P* = 0.002) and lower irisin (*P* = 0.003) than RA patients without sarcopenia. Similarly, RA patients with osteoporotic fractures (OPF) showed increased myostatin (*P* = 0.045) and decreased irisin (*P* = 0.029) compared to those without OPF. Myostatin levels correlated positively with anti-cyclic citrullinated peptide (anti-CCP) antibodies (*P* = 0.041) and inversely with body mass index (BMI) (*P* = 0.003), while irisin levels were negatively associated with C-reactive protein (*P* = 0.040) and positively linked to skeletal muscle mass index (*P* = 0.036). Multivariate analyses identified the Sharp score as an independent risk factor for OP (OR = 1.005, 95% CI 1.000–1.010; *P* = 0.04). For sarcopenia, both Sharp score (OR = 1.008, 95% CI 1.002–1.014; *P* = 0.013) and elevated myostatin levels (OR = 1.222, 95% CI 1.015–1.472; *P* = 0.034) were significant risk predictors, whereas higher irisin levels conferred protection (OR = 0.963, 95% CI 0.936–0.990; *P* = 0.007). Elevated serum myostatin and reduced irisin levels are associated with sarcopenia and OPF in RA patients. Sharp scores are identified as a risk factor for OP in RA patients. Additionally, Sharp scores and elevated myostatin levels serve as risk factors for sarcopenia, while irisin levels act as a protective factor against sarcopenia.

**Supplementary Information:**

The online version contains supplementary material available at 10.1038/s41598-025-07378-8.

## Introduction

Rheumatoid arthritis (RA), a chronic systemic autoimmune disease, is characterized by symmetrical erosive polyarthritis driven by persistent synovitis, leading to progressive cartilage and bone destruction, joint deformity, and functional disability^1^. Beyond localized joint damage, RA contributes to systemic bone loss, accelerating osteoporosis (OP)—a condition marked by reduced bone mineral density (BMD), microarchitectural deterioration, and heightened susceptibility to osteoporotic fractures (OPF)^2^. Concurrently, sarcopenia, a progressive decline in skeletal muscle mass and strength, exacerbates physical frailty, increasing risks of falls, fractures, and mortality in RA patients^3^.

**Fig. 1:**
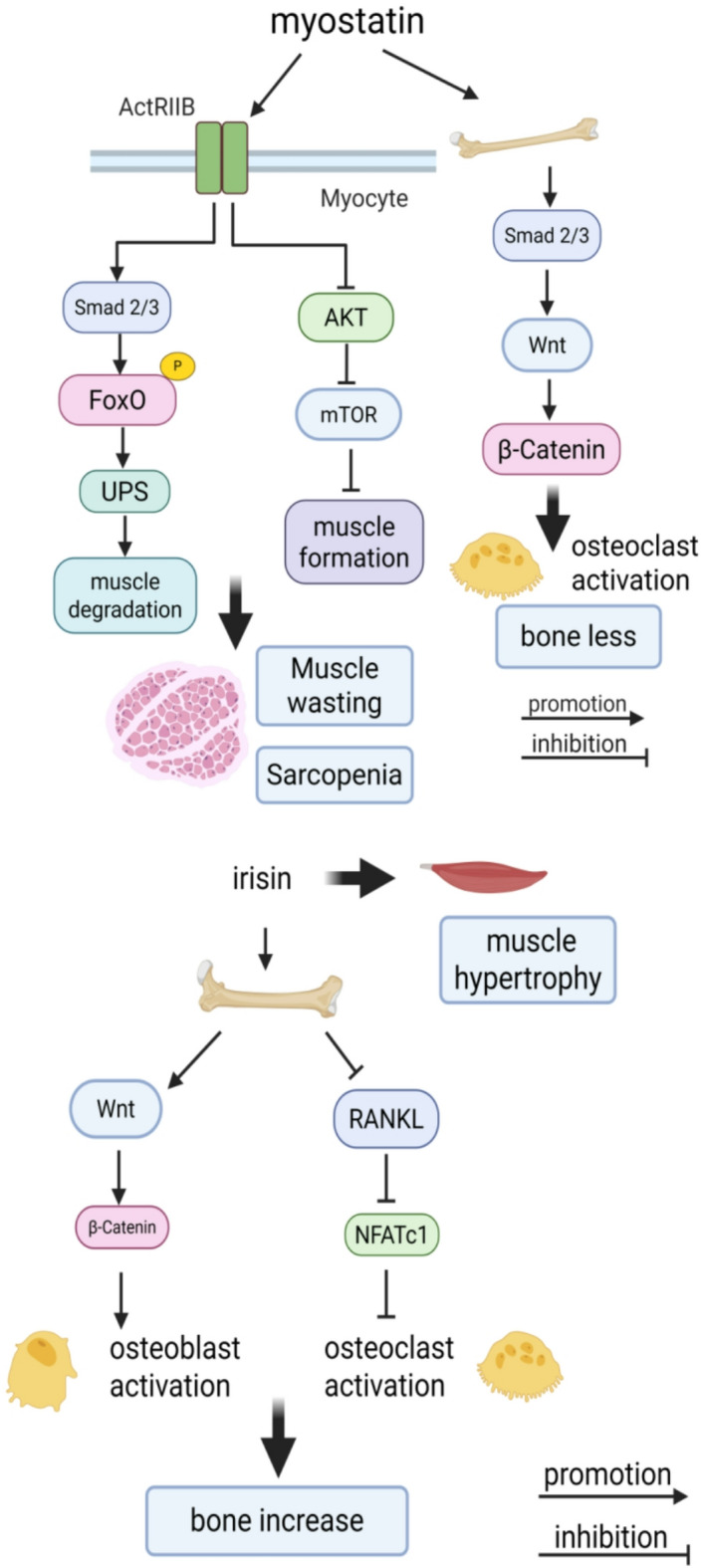
(**a**) Muscle wasting occurs through the ActRllB (activin receptor type llB) receptor, which activates the UPS (ubiquitin–proteasome system) through FoxO (fork head box transcription factors), inducing muscle degradation, and, on the other hand, inhibits the AKT (serine/threonine protein kinase)/mTOR (mammalian target of rapamycin) pathway, preventing the formation of new muscle, a condition that results in sarcopenia. Myostatin-mediated bone destruction is carried out by Smads 2/3 (mothers against decapentaplegic) activating the Wnt/β-catenin pathway that promotes the production of osteoclasts in the synovial membrane. (**b**) Increased irisin levels could promote muscle growth, while injecting irisin induced skeletal muscle hypertrophy and enhanced skeletal muscle regeneration after muscle injury. Irisin activates the Wnt/β-catenin pathway, enhances osteoblast differentiation, and promotes new bone formation. Concurrently, irisin reduces nuclear factor-kB ligand (RANKL)-induced osteoclastogenesis by inhibiting nuclear factor of activated T cells c1 (NFATc1) expression, effectively impairing osteoclasts differentiation.

Emerging evidence has established skeletal muscle as an endocrine organ, secreting cytokines termed myokines that regulate local muscle metabolism and exert systemic effects on distant tissues, including bone, adipose, brain, and liver^4^. Among these, myostatin, a transforming growth factor-β (TGF-β) superfamily member, acts as a potential negative regulator of skeletal muscle mass by inhibiting satellite cell activation and myocyte differentiation, thereby disrupting skeletal muscle regeneration^5,6^. Elevated myostatin levels increase skeletal muscle fibrosis and induce muscle atrophy, whereas the absence of myostatin induces muscle hypertrophy, as demonstrated in preclinical models^7,8^. In RA, myostatin is highly expressed in the synovial tissue and drives bone destruction via Smad-dependent activation of the Wnt/β-catenin pathway, amplifying synovial osteoclastogenesis^9,10^. Concurrently, myostatin promotes muscle wasting through ActRIIB (activin receptor type llB) receptor, which activates the UPS (ubiquitin–proteasome system) through FoxO (fork head box transcription factors), inducing muscle degradation, and, on the other hand, inhibits the AKT (serine/threonine protein kinase)/mTOR (mammalian target of rapamycin) pathway, preventing the formation of new muscle, a condition that results in sarcopenia^10^. The expression of irisin, a secreted polypeptide fragment of fibronectin type III domain-containing protein5 (FNDC5) that is hydrolyzed by proteolytic enzymes, increases in muscles following exercise^11^. Research demonstrated that increased irisin levels could promote muscle growth, while injecting irisin induced skeletal muscle hypertrophy and enhanced skeletal muscle regeneration after muscle injury^12^. Irisin participates in signal transduction pathways induced by mechanical load, downregulates osteosclerosis protein expression, activates the Wnt/β-catenin pathway in MC3T3-E1 cells, enhances osteoblast differentiation, and promotes new bone formation^13^. Concurrently, irisin reduces nuclear factor-kB ligand (RANKL)-induced osteoclastogenesis by inhibiting nuclear factor of activated T cells c1 (NFATc1) expression in RAW264.7 cells, effectively impairing osteoclasts differentiation^14^ (Fig. 1a,b).

Despite growing recognition of myokines’ therapeutic potential, critical clinical gaps persist regarding their involvement in RA-associated OP and sarcopenia. Current evidence remains predominantly preclinical, with limited studies examining circulating myostatin/irisin profiles in RA patients with OPF and sarcopenia comorbidity. This study therefore investigates serum myostatin and irisin levels in RA patients, analyzing their clinical correlations with sarcopenia and OP, particularly OPF, to identify potential biomarkers and therapeutic targets for future research.

## Materials and methods

### Study participants

**Fig. 2:**
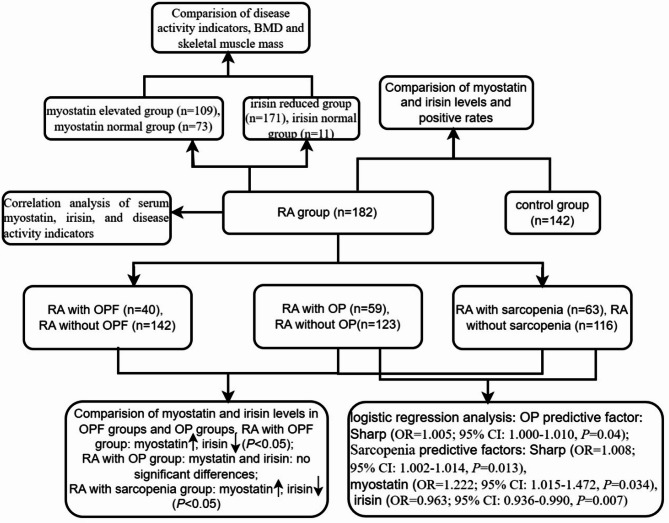
Overview of the flow chart of this study. The study employed a comparative design to investigate serum myostatin and irisin levels and positive rates in RA patients and healthy controls. The RA group was further stratified into three subgroups based on OP, OPF, and sarcopenia status for subgroup analyses of serum myostatin/irisin levels. Logistic regression models were applied to identify associations between myostatin/irisin levels and OPF/sarcopenia development. Participants were subsequently categorized by myostatin/irisin normality status to compare clinical parameters including disease activity indicators, BMD, and skeletal muscle mass. Correlation analyses were conducted to examine relationships between circulating myostatin/irisin concentrations and disease activity indicators specifically within the RA group.

The flow chart of the study is presented in Fig. 2. This cross-sectional study consecutively enrolled 182 hospitalized RA patients from the Department of Rheumatology and Immunology at the First Affiliated Hospital of Anhui Medical University (January 2018 to December 2019) and 142 age-, sex-, and body mass index (BMI) -matched healthy controls from the physical examination center. All patients fulfilled both the 1987 American College of Rheumatology (ACR) criteria and 2010 ACR/European League Against Rheumatism (EULAR) classification criteria for RA^15,16^. Power calculation using the power analysis and sample size (PASS) 2021 (v21.0.3, NCSS LLC) was based on published serum myostatin levels (RA: 3.241 ± 1.679 ng/mL vs controls: 1.717 ± 0.872 ng/mL)^17^. With α = 0.05 (two-tailed), β = 0.10, and allocation ratio 1:1, the minimum required sample size was 23 RA patients and 23 controls. Our final enrollment (RA:182; controls:142) accommodated potential 20% data attrition and preplanned subgroup analyses. To minimize potential confounding effects, strict criteria were adopted excluding chronic disorders involving vital organs (e.g. heart, liver, kidney and brain), endocrine disorders, hematological conditions, serious metabolic diseases (especially hypertension, diabetes, hypo- or hyperparathyroidism and hyperthyroidism), cancer, and acute or chronic infections. The Ethics Committee of Anhui Medical University approved the study. All procedures complied with the 1964 Helsinki Declaration and its subsequent amendments. Written informed consent was obtained from all participants.

### Clinical features

General participants’ characteristics in the RA group were recorded in detail, including age, gender, BMI, and disease duration. Clinical disease activity indices included morning stiffness, swollen joint count (SJC), tender joint count (TJC), and visual analog scale (VAS). Erythrocyte sedimentation rate (ESR), C-reactive protein (CRP), serum rheumatoid factor (RF), and anti-cyclic citrullinated peptide (anti-CCP) antibodies were measured by standard laboratory techniques. Disease activity was assessed using the 28-joint Disease Activity Score (DAS28) according to the standard formula. A Health Assessment Questionnaire (HAQ) was used to evaluate the patient’s functional status. X-ray stages (I, II, III, and IV) and Sharp scores were assessed by two radiologists using the double-blind method with the MECALL castor-50-hf model X-ray scanner for bilateral hand images. The median of glucocorticoid (GC) daily dosage was 5(10) mg, and the median of GC treatment course was 90(1780) days and the median of GC cumulative amount was 750(12, 225.0) mg*day in RA group.

### Body composition and sarcopenia assessment

Skeletal muscle mass and body composition of 182 RA patients were measured using the direct segment multi-frequency bioelectrical impedance analysis (DSM-BIA) method with the Korean InBody 720 body composition analyzer. According to the 2019 consensus of the Asian Working Group for Sarcopenia (AWGS), sarcopenia was defined as an age-related loss of muscle mass, accompanied by low muscle strength and/or impaired physical performance^18^. The 2019 AWGS cutoffs for low muscle mass in sarcopenia diagnosis were < 7.0 kg/m^2^ for men and < 5.7 kg/m^2^ for women, as measured by the BIA. Skeletal muscle mass was assessed using the BIA, while the skeletal muscle mass index (SMI) was calculated as appendicular skeletal muscle mass divided by height squared (m^2^).

### BMD measurement and diagnostic criteria for OP

BMD was measured at the hip (femoral neck and total hip) and lumbar spine (L1–L4) using dual-energy X-ray absorptiometry (DXA; GE-Lunar, DEX, Madison, WI, USA). BMD values were automatically calculated from the bone area (cm^2^) and bone mineral content (g) and expressed in g/cm^2^. According to the World Health Organization criteria, a T-score ≥  − 1 indicated normal BMD, a T-score of − 1– − 2.5 indicated osteopenia, and a T-score ≤  − 2.5 indicated OP^19^.

### Measurement of serum myostatin and irisin levels

For each subject, peripheral blood was drawn in the early morning after fasted overnight. Serum was separated and kept frozen at − 80°C until the measurement. Serum concentrations of myostatin and irisin were determined using commercially available enzyme-linked immunosorbent assay (ELISA) kits following manufacturer protocols (R&D Systems; myostatin: #DGDF80, detection range 0.2–7 ng/ml; irisin: #DY9420-50, detection range 0.5–30 ng/ml), with intra- and inter-assay coefficients of variation < 9% and < 15%, respectively.

### Statistical methods

All analyses were conducted using SPSS version 21.0 (IBM Corporation, Armonk, NY, USA). Normally distributed continuous variables were presented as mean ± standard deviation, while skewed distribution data were expressed as median with interquartile range (IQR). The two-sample t-test was used to compare continuous variables between the control and RA groups. The chi-square (χ^2^) test was used to compare inter-group rates. Non-parametric tests were used for comparisons of measurement data among multiple groups. Spearman correlation analysis was conducted to assess bivariate correlations. Multivariate logistic regression models with stepwise selection were constructed to identify predictors of OP and sarcopenia, adjusting for clinically relevant confounders (age, sex, and BMI). Effect estimates are reported as odds ratios (ORs) with 95% confidence intervals (CIs). All tests were two-tailed with statistical significance defined as *P* < 0.05.

## Results

### Demographic characteristics and comparison of serum myostatin and irisin levels between RA and control groups

This study comprised 182 RA patients aged 20–89 years (female: 78.0%, mean age: 57 ± 13 years, BMI: 22 ± 4 kg/m^2^) and 142 age-, sex-, and BMI-matched controls (female: 78.2%, mean age: 57 ± 10 years, BMI: 23 ± 4 kg/m^2^), demonstrating successful demographic matching (all *P* > 0.05 by Mann–Whitney U test). Homogeneity of variances was assessed using Levene’s test. As the assumption of equal variances was violated (*P* < 0.05), non-parametric comparisons was employed for group comparisons (between RA and control groups). Serum myostatin levels were significantly higher in RA patients than in healthy controls (5.41 [3.99–6.41] vs. 4.74 [3.93–5.18] ng/mL, *P* = 0.001). Serum irisin levels were significantly lower in RA patients compared to controls (31.10 [24.44–37.89] vs. 66.89 [25.27–86.02] ng/mL, *P* = 0.009). Receiver operating characteristic (ROC) analysis established optimal biomarker thresholds for RA discrimination (Supplementary figures S1 and S2). The area under the curve (AUC) values were 0.604 for myostatin and 0.584 for irisin (*P* < 0.05). Participants were categorized into two groups based on baseline cutoff values of myostatin (5.12 ng/mL) and irisin (58.04 ng/mL): myostatin normal and myostatin elevated groups, and irisin normal and irisin reduced groups. Additionally, the positive rates of elevated myostatin and reduced irisin were significantly higher in RA patients than in controls (myostatin: 59.9% vs. 25.4%, χ^2^ = 38.484, *P* < 0.001; irisin: 94.0% vs. 43.0%, χ^2^ = 1022.033, *P* < 0.0001) (Table 1).

**Table 1:** Demographic characteristics and comparison of myostatin and irisin levels in RA and control groups.

Variables	RA (n = 182)	Control (n = 142)	*P* value
Age, mean (SD), years	57 ± 13	57 ± 10	0.937
Female, % (n)	78.0% (142)	78.2% (111)	0.975
BMI, mean (SD), kg/m^2^	22 ± 4	23 ± 4	0.916
Myostatin, M(P_25_–P_75_), ng/mL	5.41 (3.99–6.41)	4.74 (3.93–5.18)	0.001
Irisin, M(P_25_–P_75_), ng/mL	31.10 (24.44–37.89)	66.89 (25.27–86.02)	0.009
Myostatin, % (n)	59.9% (109)	25.4% (36)	< 0.0001
Irisin, % (n)	94.0% (171)	43.0% (61)	< 0.0001

Statistical significance *P* < 0.05.

*P* value presents statistical significance between control group and RA group.

*BMI* body mass index.

### Comparison of disease activity indicators among myostatin and irisin groups in RA patients

Given the unequal sample sizes between myostatin/irisin normal and abnormal groups in RA patients, Mann–Whitney U tests (for non-parametric comparisons) were employed to compare continuous variables in non-normally distributed variable. No significant differences were observed in SJC, TJC, morning stiffness, VAS, ESR, CRP, DAS28, and Sharp scores between normal and elevated myostatin groups (all *P* > 0.05). Similarly, these disease activity indicators also showed no significant differences between normal and reduced irisin groups (all *P* > 0.05) (Supplementary table S1).

### Comparison of BMD at different sites among myostatin and irisin groups in RA patients

Among myostatin and irisin different groups, the BMD at the lumbar spine (L1, L2, L3, L4, and L1-L4) and hip (neck, Ward’s triangle, greater trochanter, and whole hip) were compared, respectivily. BMD at the L1 in the elevated myostatin group was significantly lower than in the normal myostatin group (*P* = 0.038), while other sites demonstrated no significant differences (all *P* > 0.05). Additionally, BMD at different sites showed no significant differences between normal and reduced irisin groups (all *P* > 0.05) (Supplementary table S2).

### Comparison of skeletal muscle mass at different anatomical sites and SMI among myostatin and irisin groups in RA patients

Skeletal muscle mass at any site and SMI showed no significant differences between elevated myostatin and normal myostatin groups in RA patients (all *P* > 0.05). In contrast, RA patients in the reduced irisin group exhibited significantly lower skeletal muscle mass at all sites and a lower SMI compared with those in the normal irisin group (all *P* < 0.05) (Table 2).

**Table 2:** Comparison of skeletal muscle parameters and SMI in RA patients of different myostatin and irisin groups (M [P25, P75]).

Position of muscle mass (g)	Myostatin elevated group (n = 109)	Myostatin normal group (n = 73)	*P*1 value	Irisin reduced group (n = 171)	Irisin normal group (n = 11)	*P*2 value
Right upper limb	1.67 (1.39–2.00)	1.71 (1.45–2.14)	0.220	1.66 (1.39–2.01)	2.07 (1.66–2.78)	0.014
Left upper limb	1.63 (1.37–1.97)	1.70 (1.44–2.11)	0.196	1.65 (1.38–2.01)	2.09 (1.68–2.94)	0.012
Right lower limb	5.31 (4.77–6.07)	5.37 (4.76–6.20)	0.761	5.30(4.75–6.10)	6.72 (5.67–8.83)	0.002
Left lower limb	5.33 (4.69–5.93)	5.44 (4.75–6.27)	0.387	5.32 (4.69–5.94)	6.85 (5.90–8.83)	< 0.0001
Trunk	15.96 (14.35–17.80)	16.38 (14.96–18.75)	0.247	16.06 (14.39–17.90)	18.49 (16.16–22.8)	0.020
Skeletal muscle mass	33.9 (30.80–37.30)	33.5 (30.9–38.1)	0.786	33.7 (30.6–36.8)	41 (33.5–51)	0.003
SMI (g/m^2^)	5.51 (4.99–6.20)	5.91 (5.06–6.47)	0.388	5.57 (5.00–6.23)	6.71 (6.20–7.37)	< 0.0001

Statistical significance *P* < 0.05.

*P*1 value indicates statistical significance between elevated myostatin and myostatin normal groups.

*P*2 value indicates statistical significance between reduced irisin and irisin normal groups.

*SMI* skeletal muscle index.

### Serum myostatin and irisin levels were compared between OP, OPF and sarcopenia groups in RA patients

Among 182 RA patients, there were no statistically significant differences in serum myostatin and irisin levels between RA patients with and without OP (all *P* > 0.05). Myostatin levels were significantly higher in RA patients with OPF compared to those without OPF (*P* = 0.045). Concurrently, serum irisin levels were significantly lower in RA patients with OPF compared to those without OPF (*P* = 0.029). Among 182 RA patients (with three missing cases), myostatin levels were significantly higher in RA patients with sarcopenia than in RA patients without sarcopenia (*P* = 0.002). Similarly, irisin levels were significantly lower in RA patients with sarcopenia than in RA patients without sarcopenia (*P* = 0.003) (Table 3).

**Table 3:** Comparison of serum myostatin and irisin levels in OP, OPF and sarcopenia groups in RA patients (M [P25, P75]).

Groupings	Cases	Myostatin (ng/mL)	Irisin (ng/mL)
RA with OP	59	5.41 (4.18–6.37)	31.19 (24.91–38.12)
RA without OP	123	5.42 (3.86–6.46)	30.87 (24.12–37.78)
z1		0.024	0.597
*P*1		0.981	0.551
RA with OPF	40	5.50 (4.39–6.46)	24.86 (19.74–36.32)
RA without OPF	142	4.66 (3.11–6.08)	31.19 (24.60–37.98)
z2		2.007	2.189
*P*2		0.045	0.029
RA with sarcopenia	116	5.56 (4.54–6.55)	28.78 (23.23–36.00)
RA without sarcopenia	63	4.68 (3.26–5.93)	32.36 (26.86–43.90)
z3		3.042	2.964
*P*3		0.002	0.003

Statistical significance *P* < 0.05.

*P*1 indicates statistical significance between RA with and without OP groups.

*P*2 indicates statistical significance between RA with and without OPF groups.

*P*3 indicates statistical significance between RA with and without sarcopenia groups.

*RA* Rheumatoid arthritis, *OP* osteoporosis, *OPF* Osteoportic fracture.

### Correlation analysis of serum myostatin, irisin, and disease activity indicators in RA patients

In 182 RA patients, serum myostatin levels were negatively correlated with BMI (r =  − 0.220, *P* = 0.003) and positively correlated with CCP (r = 0.152, *P* = 0.041). Irisin was positively correlated with SMI (r = 0.156, *P* = 0.036) and negatively correlated with CRP (r =  − 0.153, *P* = 0.040). Other disease activity indicators showed no statistically significant correlations (*P* > 0.05) (Table 4). No significant correlations were observed between serum myostatin/irisin and other indicators (e.g., right/left upper limb muscle mass, right/left lower limb muscle mass, trunk muscle mass, skeletal muscle and BMD-hip/L1/L2/L3/L4/L1-L4) in RA patients (supplementary table S3).

**Table 4:** Correlation of serum myostatin and irisin levels with disease indicators in RA (rs [*P*]).

Disease activity indicators	Myostatin (ng/mL)	Irisin (ng/mL)
SJC	0.065 (0.181)	0.017 (0.819)
TJC	0.824 (0.181)	0.028 (0.706)
VAS	− 0.005 (0.946)	0.039 (0.603)
HAQ	0.042 (0.573)	0.071 (0.345)
Morning stiffness	− 0.045 (0.552)	− 0.044 (0.558)
Joint function	0.065 (0.384)	0.122 (0.103)
ESR (mm/h)	0.086 (0.249)	− 0.077 (0.302)
CRP (mg/L)	− 0.037 (0.616)	− 0.153 (0.040)
RF (IU/mL)	0.004 (0.956)	− 0.004 (0.956)
Anti-CCP (RU/mL)	0.152 (0.041)	− 0.020 (0.791)
DAS28	0.077 (0.309)	0.011 (0.882)
BMI (kg/m^2^)	− 0.220 (0.003)	0.056 (0.454)
SMI (g/m^2^)	− 0.070 (0.354)	0.156 (0.036)
X-ray stages	0.103 (0.166)	0.012 (0.869)
Sharp scores	0.087 (0.243)	0.025 (0.741)

Statistical significance *P* < 0.05.

*STC* swollen joint count, *TJC* tender joint count, *VAS* visual analogue scale, *HAQ* Health Assessment Questionnaire *ESR* erythrocyte sedimentation rate, *CRP* C-reactive protein, *RF* rheumatoid factor, anti-*CCP* anti-cyclic citrullinated peptide antibodies, *DAS28* 8-joint Disease Activity Score, *BMI* body mass index, *SMI* skeletal muscle index, *BMD* bone mineral density.

### Logistics regression analysis of OP and sarcopenia in RA patients

Binary logistic regression (stepwise) analyses were conducted to identify potential risk and protective factors associated with OP and sarcopenia in RA patients. In RA group, OP was used as the dependent variable (0 = no OP, 1 = OP) and sarcopenia was defined as the dependent variable (0 = no sarcopenia, 1 = sarcopenia). Univariate logistic regression analyses were performed for all independent variables (VAS, HAQ, morning stiffness, CRP, RF, anti-CCP, Sharp scores, DAS28, disease duration, GC use, and serum myostatin and irisin levels), respectively, and followed by multivariate logistic regression analyses adjusted for age, sex, and BMI. Sharp scores (OR = 1.005; 95% CI: 1.000–1.010; *P* = 0.04) were significant risk factors for OP in RA patients. Sharp scores (OR = 1.008; 95% CI: 1.002–1.014; *P* = 0.013) and higher serum myostatin levels (OR = 1.222; 95% CI: 1.015–1.472; *P* = 0.034) were significant risk factors for sarcopenia in RA patients. Conversely, irisin levels (OR = 0.963; 95% CI: 0.936–0.990; *P* = 0.007) were protective factors for sarcopenia in RA patients (Tables 5, 6).

**Table 5:** Binary logistic regression analysis to identify OP complexity in RA patients.

Variables	Univariate analysis		Multivariate analysis	
	Crude OR (95%CI)	*P* value	Adj. OR (95%CI)	*P* value
VAS	0.945 (0.811–1.100)	0.463	0.967 (0.800–1.169)	0.728
HAQ	0.995 (0.884–1.120)	0.935	1.022 (0.856–1.220)	0.811
Morning stiffness	1.001 (0.996–1.006)	0.628	1.003 (0.997–1.009)	0.305
CRP (mg/L)	0.992 (0.984–1.001)	0.099	0.992 (0.982–1.003)	0.160
RF (IU/mL)	1.000 (0.999–1.001)	0.858	1.001 (0.999–1.002)	0.284
Anti-CCP (RU/mL)	1.000 (1.000–1.001)	0.172	1.000 (1.000–1.001)	0.225
Sharp scores	1.005 (1.001–1.009)	0.012	1.005 (1.000–1.010)	0.040
DAS28	1.003 (0.758–1.328)	0.981	1.107 (0.780–1.571)	0.569
Disease duration	1.034 (1.001–1.068)	0.042	1.020 (0.980–1.062)	0.327
GC use	0.979 (0.520–1.844)	0.947	0.852 (0.382–1.897)	0.694
Myostatin (ng/mL)	0.942 (0.829–1.071)	0.361	0.987 (0.825–1.182)	0.890
Irisin (ng/mL)	0.999 (0.984–1.016)	0.943	1.010 (0.988–1.033)	0.354

*P* significant at < 0.05.

Multivariate logistic regression models were adjusted for age, sex, and BMI.

*BMI* body mass index *CRP* C-reactive protein, *RF* rheumatoid factor, anti-*CCP* anti-cyclic citrullinated peptide antibodies, *DAS28* 8-joint Disease Activity Score *VAS* visual analogue scale, *HAQ* Health Assessment Questionnaire *GC* Glucocorticoid.

**Table 6:** Binary logistic regression analysis to identify sarcopenia complexity in RA patients.

Variables	Univariate analysis		Multivariate analysis	
	Crude OR (95% CI)	*P* value	Adj. OR (95% CI)	*P* value
VAS	1.060 (0.913–1.232)	0.443	1.0077 (0.902–1.286)	0.410
HAQ	1.255 (0.851–1.851)	0.252	1.235 (0.775–1.967)	0.374
Morning stiffness	0.999 (0.994–1.003)	0.342	0.999 (0.994–1.005)	0.809
CRP (mg/L)	0.999 (0.991–1.006)	0.707	0.999 (0.990–1.007)	0.750
RF (IU/mL)	1.000 (0.990–1.001)	0.460	1.000 (0.998–1.001)	0.569
Anti-CCP (RU/mL)	1.001 (1.000–1.002)	0.068	1.001 (1.000–1.002)	0.066
Sharp scores	1.009 (1.004–1.014)	0.001	1.008 (1.002–1.014)	0.013
DAS28	1.177 (0.892–1.554)	0.250	1.162 (0.841–1.606)	0.362
Disease duration	1.037 (1.002–1.074)	0.040	1.044 (0.998–1.093)	0.061
GC use	1.093 (0.585–2.044)	0.778	1.111 (0.521–2.268)	0.786
Myostatin (ng/mL)	1.286 (1.076–1.537)	0.006	1.222 (1.015–1.472)	0.034
Irisin (ng/mL)	0.965 (0.942–0.988)	0.003	0.963 (0.936–0.990)	0.007

*P* significant at < 0.05.

Multivariate logistic regression models were adjusted for age, sex, and BMI.

*BMI* body mass index *CRP* C-reactive protein, *RF* rheumatoid factor, anti-*CCP* anti-cyclic citrullinated peptide antibodies, *DAS28* 8-joint Disease Activity Score *VAS* visual analogue scale, *HAQ* Health Assessment Questionnaire *GC* Glucocorticoid.

## Discussion

RA, an immune-mediated inflammatory disorder, manifests as persistent synovial inflammation that drives progressive joint destruction and systemic bone loss, ultimately contributing to OP development. This chronic condition not only causes debilitating pain but also significantly impairs quality of life and functional capacity, often resulting in work disability. OP, characterized by increased bone fragility due to the destruction of bone tissue microarchitecture, remains one of the most common complications of RA^20^. Tong JJ et al. found that OP incidence in RA patients was more than twice that of the healthy population^21^. Furthermore, sarcopenia is a notable comorbidity of RA that may be considered its extra-articular manifestation, affecting approximately 25% of RA patients^22^. Studies also found that sarcopenia prevalence in RA patients is higher than in healthy people^23^. Although myokines are recognized as key pro-inflammatory cytokines in various diseases, their role involvement in RA-associated OP and sarcopenia progression remains unclear. To address this knowledge gap, our study aimed to investigate the clinical correlations between serum myostatin/irisin levels and sarcopenia and OP in RA patients.

Myostatin, a TGF-β superfamily member and growth differentiation factor (GDF-8), is primarily found in the blood and is highly expressed in skeletal muscles. It inhibits muscle growth and differentiation by binding to ACVRIIB^10^. A Chinese cohort study by Lin et al. (n = 344 RA vs. 118 controls) demonstrated markedly elevated baseline serum myostatin levels in RA patients (3.241 ± 1.679 ng/mL) versus healthy individuals (1.717 ± 0.872 ng/mL; *P* < 0.001)^17^. Similarly, our results showed that serum myostatin levels in 182 RA patients were significantly higher than in controls (*P* = 0.001). Notably, the myostatin positive rate in RA patients was 59.9% (109/182) compared to 25.4% (36/142) in the control group, which was 2.36 times higher (*P* < 0.0001). Murillo-saich JD et al. found a lower SMI (*P* = 0.008) and a higher myostatin concentration in 84 RA patients compared to 127 controls (9.0 [1.2–140] ng/mL vs. 3.5 [1.0–89.9] ng/mL, *P* < 0.001)^24^. These findings are consistent with our results. Additionally, serum myostatin levels were directly correlated with RA duration (13 [0–45] years) (r = 0.24, *P* = 0.02), CRP (r = 0.48, *P* < 0.001), ESR (r = 0.28, *P* = 0.009), and DAS28-ESR (r = 0.22, *P* = 0.04) and were negatively correlated with SMI (r =  − 0.29, *P* = 0.008). Multiple logistic regression analysis revealed that myostatin was associated with disease activity in RA patients (*P* = 0.027)^24^. Our study found no significant differences in SJC, TJC, morning stiffness, VAS, DAS28, CRP, SADI, CDAI, SMI, and Sharp scores between elevated myostatin and normal myostatin groups. Additionally, myostatin and these disease activity indicators showed no significant correlation in RA patients. The differences between our results and those of previous studies may be attributed to inconsistencies in the selected ethnic populations, grouping criteria, and detection methods for relevant indicators. However, myostatin was positively correlated with anti-CCP levels (r = 0.152, *P* = 0.041) in our study. Myostatin is known to activate the phosphatidylinositol 3‐kinase (PI3K)-Akt signaling pathway and interleukin (IL)-1β pathway in RA synovial fibroblasts, inducing pro-inflammatory cytokines such as tumor necrosis factor‐α (TNF-α) and IL-6^9,25^. These cytokines upregulate peptidylarginine deiminase (PAD) enzymes, which catalyze citrullination of proteins, generating antigens targeted by anti-CCP antibodies^26^. Thus, myostatin may indirectly promote anti-CCP antibody production by amplifying the inflammatory microenvironment that drives citrullinated antigen exposure.

Irisin is a myokine produced by skeletal muscle in response to exercise^27^. Injecting irisin into mice induces skeletal muscle hypertrophy by upregulating the expression of related genes; thus, irisin promotes muscle growth^12^. Lavrova et al. measured serum irisin levels using ELISA in 170 subjects, including 110 RA patients and 60 healthy individuals. They identified an association between lower irisin concentrations and higher RA activity degree (DAS28), extra-articular manifestations, greater joint dysfunction, longer disease duration (5–10 years), and lower 25(OH)D levels^28^. Yu Yongmei et al. examined serum irisin levels in 46 RA patients and 33 healthy individuals. They showed that serum irisin levels were significantly lower in RA patients than in controls and were negatively correlated with anti-CCP, ESR, and DAS28 levels^29^. Similar findings were observed in our study. Irisin levels were significantly lower in 182 RA patients than in controls (*P* = 0.009), while the positive irisin rate was significantly higher in RA patients compared to controls (94% vs. 43%, χ^2^ = 102.033, *P* < 0.0001), being approximately 2.19 times higher. While our study detected no significant differences in SJC, TJC, morning stiffness, VAS, DAS28, SADI, CDAI, and Sharp scores between irisin reduced and normal groups. Furthermore, we identified no significant correlation between irisin and disease activity indicators in RA patients. However, irisin was negatively correlated with CRP (r =  − 0.153, *P* = 0.040). Therefore, lower irisin levels are associated with higher CRP levels, which is consistent with the findings of previous studies. Accordingly, further research is warranted to validate these results given the limited number of clinical studies on irisin in RA patients.

OP is a major cause of fractures in RA patients and is characterized by decreased bone mass and microstructural degeneration of bone tissue. Wu et al. collected data from 6,308 elderly Chinese subjects, measuring plasma myostatin, BMD, and bone metabolism indices. After adjusting for age and sex, they found a positive correlation of myostatin with lean body mass (LBM) (b = 0.51, *P* < 0.001)^30^. Determining the levels of mature myostatin and calculating the relative abundance of mature myostatin (mature myostatin/total myostatin), which was approximately 54% ± 2.4%, were necessary to further investigate the effect of myostatin on BMD. High and low BMD groups demonstrated no significant difference in mature myostatin levels ([1.69 ± 0.08] vs. [1.79 ± 0.11] ng/ml). The relative abundance of mature myostatin in the high BMD group was significantly lower compared to the low BMD group (49.0% ± 4.1%] vs. [59.5% ± 2.5%], *P* = 0.021). Hence, the relative abundance of mature myostatin was negatively correlated with BMD in the Chinese elderly population. In our study, L1 BMD was lower in RA patients with elevated myostatin levels than in patients with normal myostatin levels (*P* = 0.038), but no significant differences were observed in BMD at other sites. This discrepancy may be attributed to differences in study populations, sample size and grouping methods, which could have influenced the results. Our subgroup analysis revealed that RA patients with and without OP showed no significant difference in myostatin levels (*P* > 0.05). However, RA patients with OPF exhibited significantly higher myostatin levels compared to RA patients without OPF (*P* = 0.045). Mechanistically, this pathologically elevated myostatin may induce bone destruction by coordinately activating the Smad2/3-Wnt/β-catenin signaling pathways, ultimately promoting excessive osteoclastogenesis within the synovial microenvironments^9,10^. In addition, Gamez-Nava et al. and Palermo et al. found that low irisin levels were negatively correlated with OPF^31,32^. Serum irisin levels in the fracture group were lower than in controls ([0.69 ± 0.22] vs. [0.80 ± 0.20] μg/mL, *P* = 0.032). Serum irisin and BMD demonstrated no significant correlation at the whole hip (r = 0.083, *P* = 0.489), femoral neck (r = 0.135, *P* = 0.256), or lumbar spine (r = 0.070, *P* = 0.560). The results confirm an inverse correlation between irisin levels and vertebral fragility fractures in postmenopausal women with OPF, suggesting that irisin may play a protective role in bone health independent of BMD^33^. Analogously, our study found that 182 RA patients exhibited no significant differences in BMD of the lumbar spine (L1, L2, L3, L4, and L1-L4) and hip (neck, Ward’s triangle, greater trochanter, and whole hip) across different irisin groups. Notably, RA patients with OPF exhibited significantly reduced serum irisin levels compared to those without OPF (*P* = 0.029). These results are consistent with the well-documented inverse relationship between irisin and OPF progression, suggesting its potential protective role in bone integrity. Mechanistically, irisin exerts dual osteoprotective effects by activating the Wnt/β-catenin signaling pathway to stimulate osteoblast differentiation and bone formation, and suppressing RANKL-induced osteoclastogenesis through inhibition of NFATc1 nuclear translocation^13,14^. This coordinated regulation of osteoblast-osteoclast coupling creates a bone remodeling equilibrium that likely mitigates fracture risk in RA patients.

Sarcopenia is a prevalent comorbidity in RA. A cross-sectional study of 388 Japanese women with RA reported a sarcopenia prevalence of 37% based on the AWGS criteria^33^. However, this estimate increased to 51% among participants aged ≥ 65 years, substantially higher than that observed in healthy Japanese women of the same age^33^. Foreign studies also reported that the prevalence of sarcopenia ranges from 3 to 24%, depending on the diagnostic criteria used, and increases with age^34^. Additionally, 20%–30% of patients with RA exhibit sarcopenia, which is associated with disease severity^34^. Di Monaco et al. conducted a study involving 313 elderly women, finding that 180 participants exhibited reduced skeletal muscle mass, resulting in a 57.5% sarcopenia incidence^35^. Similarly, our study demonstrated that sarcopenia incidence in RA patients was 64.8%. The pathogenesis of sarcopenia is multifactorial, encompassing factors such as aging, physical inactivity, neuromuscular injury, endocrine dysregulation, oxidative stress, and inflammation. Sarcopenia can significantly impair bodily function, cause metabolic disorders, increase morbidity and mortality, incur higher medical costs, and lead to serious consequences. Currently, no specific biomarkers have been established for diagnosing sarcopenia, necessitating further research and exploration. Sarcopenia treatment requires a comprehensive approach, including adequate protein and fatty acid intake, regular physical exercise, and the use of anti-inflammatory medications. Selective androgen receptor modulators and anti-myostatin antibodies represent potential stimulators of muscle anabolism^34^. Su CM et al. found a positive correlation between myostatin and TNF‐α, a well‐known proinflammatory cytokine, in RA synovial tissue and indicated that myostatin increases TNF‐α expression via the PI3K–Akt–AP‐1 signaling pathway in human RA synovial fibroblasts^9^. Our results demonstrated that serum myostatin levels were significantly higher in RA patients with sarcopenia compared to those without sarcopenia (*P* = 0.002). Conversely, irisin levels were significantly lower in RA patients with sarcopenia than in those without sarcopenia (*P* = 0.003). In the myostatin group, no significant differences in skeletal muscle mass or SMI levels were observed across all regions of RA patients between the two groups (*P* > 0.05). In contrast, both skeletal muscle mass and SMI levels were significantly lower in reduced irisin subgroup compared to the normal irisin subgroup (*P* < 0.05). Regression analysis revealed that Sharp scores and elevated myostatin levels were risk factors for sarcopenia in RA patients, whereas irisin served as a protective factor. This may be attributed to the negative regulatory effect of myostatin on muscle growth and its significant impact on skeletal muscle repair. Specifically, myostatin inhibits the activation of satellite cells and muscle cell differentiation and transformation, thereby disrupting skeletal muscle regeneration. Sarcopenia is primarily associated with the reduction in skeletal muscle mass and function^6,36^. Thus, elevated serum myostatin levels contribute to the degradation of skeletal muscle, ultimately leading to sarcopenia. This underscores the role of myostatin as a risk factor for sarcopenia. Irisin primarily regulates muscle growth–related genes via the extracellular signal-regulated kinase (ERK) pathway, promoting muscle growth. Consequently, lower irisin levels are associated with reduced skeletal muscle mass, increasing the likelihood of developing sarcopenia^37^. Irisin, a myokine induced by exercise, antagonizes myostatin’s effects by promoting anti-inflammatory pathways (e.g., AMPK activation). Reduced irisin levels in RA patients may amplify myostatin-driven inflammation and muscle atrophy^38,39^. Therefore, irisin serves as a protective factor against sarcopenia development.

The limitations of our study include reduced generalizability, a relatively small sample size (particularly the subgroups with the RA group). Participants were recruited from the First Affiliated Hospital of Anhui Medical University, An Hui provinces in China. Therefore, it is not sufficient to represent Chinese patients. Additionally, our study lacks direct data on exercise habits and nutritional intake-key determinants of irisin levels, which may influence the results and thus will be integrated objective measures (e.g., accelerometry for physical activity, 24-h dietary recalls) to better control these confounders in future studies. While the cross-sectional design limits causal interpretation of the relationships between serum myostatin/irisin with sarcopenia/OP in Chinese RA patients. Future studies should include dynamic follow-up and multifactor analysis. Subsequent investigations should prioritize elucidating the specific mechanism pathway and evaluating translational potential through multicenter cohorts and multi-omics integration trials. The novelty of our research lies in its innovative topic selection and thesis ideas as few researchers have explored this area both domestically and internationally.

## Conclusions

Serum myostatin levels were significantly elevated, while irisin levels were significantly reduced in RA patients. RA patients with sarcopenia had higher myostatin and lower irisin than RA patients without sarcopenia. Similarly, RA patients with OPF showed increased myostatin and decreased irisin compared to those without OPF. In conclusion, myostatin and irisin are significantly correlated with sarcopenia and OPF in RA patients. Additionally, Sharp scores and elevated myostatin levels serve as risk factors for sarcopenia, whereas irisin levels act as a protective factor against sarcopenia in RA patients.

## Electronic supplementary material

Below is the link to the electronic supplementary material.

Supplementary Material 1

Supplementary Material 2

## Data Availability

All data are included in the manuscript in the tables.
