# Supplementary material for: Correlations of serum myostatin and irisin with sarcopenia and osteoporosis in rheumatoid arthritis patients: a cross-sectional study
Source: Sci Rep. 2025 Jul 2;15:23068. doi: 10.1038/s41598-025-07378-8 (PMC12216405; doi:10.1038/s41598-025-07378-8)
Supplement: Supplementary file 2 — Supplementary Material 2 [file 41598_2025_7378_MOESM2_ESM.docx]

**Supplementary tables:**

Table S1. Comparison of disease activity indicators in RA patients of different myostatin and irisin groups (M [P25, P75])

| Disease activity  indicators | Myostatin elevated group  (n=109) | Myostatin  normal group  (n=73) | *P*1  value | Irisin reduced  group  (n=171) | Irisin normal group  (n=11) | *P*2  value |
| --- | --- | --- | --- | --- | --- | --- |
| SJC | 2 (1–6) | 1 (0–6) | 0.100 | 2 (1–6) | 3(1–4.75) | 0.551 |
| TJC | 8(3.5–13) | 7.5 (2.25–12.75) | 0.390 | 8 (3–13) | 6.5(4–11.25) | 0.623 |
| Morning stiffness | 5 (0–30) | 5(0–30) | 0.998 | 5(0–30) | 1 (0–15) | 0.131 |
| VAS | 6 (4–7) | 6 (4–8) | 0.763 | 6 (4–7) | 7 (4–8) | 0.540 |
| ESR (mm/h) | 54 (37–81.75) | 48 (27.75–74.5) | 0.199 | 51(33–79) | 81 (44–91) | 0.237 |
| CRP (mg/L) | 21.4(7.08–45.5) | 21.1 (8.4–62.65) | 0.556 | 21.15 (7.23–54.43) | 31.5 (10.6-76.7) | 0.279 |
| DAS28 | 4.92(4.12–5.65) | 4.54 (3.82–5.44) | 0.120 | 4.75 (4.06–5.58) | 4.85 (4.27–5.63) | 0.839 |
| Sharp scores | 47 (13.5–125.5) | 45(8–1119.5) | 0.400 | 46 (12–126) | 45 (28–104) | 0.768 |

Statistical significance *P* < 0.05

*P*1 value indicates statistical significance between myostatin elevated and myostatin normal groups

*P*2 value indicates statistical significance between irisin reduced and irisin normal groups

*STC* swollen joint count, *TJC* tender joint count, *VAS* visual analogue scale, *ESR* erythrocyte sedimentation rate, *CRP* C-reactive protein, *DAS28* 28-joint disease activity score

Table S2. Comparison of BMD in the lumbar spine and hip in RA patients of different myostatin and irisin groups (M [P25, P75])

| BMD  (g/cm^2^) | Myostatin elevated  group (n=109) | Myostatin  normal  group (n=73) | *P*1  value | Irisin  reduced  group  (n=171) | Irisin  normal  group  (n=11) | *P*2  value |
| --- | --- | --- | --- | --- | --- | --- |
| Neck | 0.78(0.68–0.88) | 0.77 (0.68–0.87) | 0.784 | 0.78(0.68–0.88) | 0.73(0.64–0.88) | 0.777 |
| Ward's triangle | 0.62(0.51–0.72) | 0.60(0.50–0.72) | 0.732 | 0.61(0.51–0.72) | 0.63 (0.51–0.72) | 0.939 |
| greater trochanter | 0.64(0.55–0.71) | 0.63 (0.54–0.76) | 0.875 | 0.63(0.54–0.73) | 0.69 (0.56–0.76) | 0.512 |
| Hip | 0.82(0.72–0.91) | 0.83 (0.73–0.93) | 0.518 | 0.82(0.72–0.92) | 0.87 (0.69–0.94) | 0.699 |
| L1 | 0.85(0.77–0.97) | 0.92 (0.79–1.00) | 0.038 | 0.88(0.77– 0.98) | 0.86 (0.77–1.01) | 0.820 |
| L2 | 0.89(0.78–1.04) | 0.95 (0.84–1.05) | 0.170 | 0.91(0.80–1.04) | 1.00 (0.85–1.05) | 0.417 |
| L3 | 0.96(0.87–1.11) | 1.02 (0.91–1.14) | 0.177 | 0.99(0.88–1.13) | 1.09(0.89–1.11) | 0.557 |
| L4 | 0.99(0.90–1.15) | 1.04 (0.90–1.16) | 0.681 | 0.99(0.89–1.13) | 1.09 (0.91–1.17) | 0.366 |
| L1-L4 | 0.93(0.84–1.06) | 0.99 (0.89–1.07) | 0.218 | 0.95 (0.85–1.07) | 1.09 (0.98–1.12) | 0.335 |

Statistical significance *P* < 0.05

*P*1 value indicates statistical significance between myostatin elevated and myostatin normal groups

*P*2 value indicates statistical significance between irisin reduced and irisin normal groups

*BMD* bone mineral density *L* lumbar

Table S3. Correlation of myostatin and irisin levels with muscle mass and BMD in RA (rs [*P*])

| Disease activity indicators | Myostatin (ng/ml) |  | Irisin (ng/ml) |
| --- | --- | --- | --- |
| Right upper limb muscle mass (g)  Left upper limb muscle mass (g)  Right lower limb muscle mass (g)  Left lower limb muscle mass (g)  Trunk muscle mass (g)  Skeletal muscle mass (g)  BMD-hip (g/cm^2^)  BMD-L1 (g/cm^2^)  BMD-L2 (g/cm^2^)  BMD-L3 (g/cm^2^)  BMD-L4 (g/cm^2^)  BMD-L1-L4 (g/cm^2^) | −0.104 (0.164)  −0.104 (0.162)  −0.017 (0.823)  −0.059 (0.432)  −0.102 (0.170)  −0.030 (0.683)  −0.037 (0.622)  −0.116(0.120)  −0.050 (0.499)  −0.053 (0.474)  −0.042 (0.570)  −0.053 (0.482) |  | 0.066 (0.376)  0.065 (0.385)  −0.113 (0.129)  −0.122 (0.100)  −0.077 (0.299)  −0.100 (0.181)  −0.088 (0.236)  −0.064 (0.394)  −0.079 (0.289)  −0.020 (0.786)  −0.076 (0.307)  −0.052 (0.484) |

Statistical significance *P* < 0.05

*L* lumbar

**Supplementary figures:**


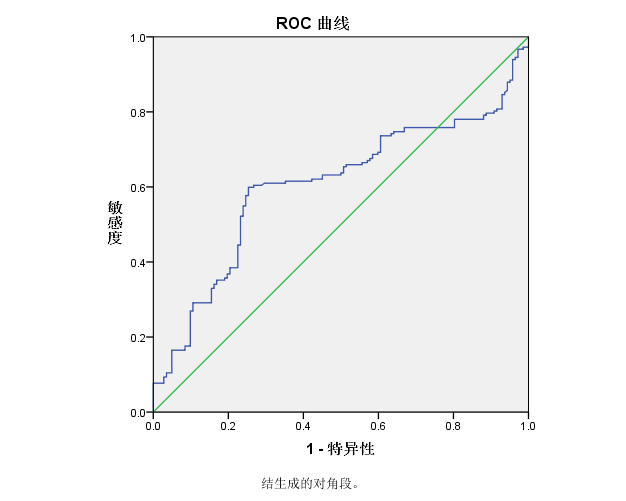


Figue S1 ROC curve of serum myostatin levels


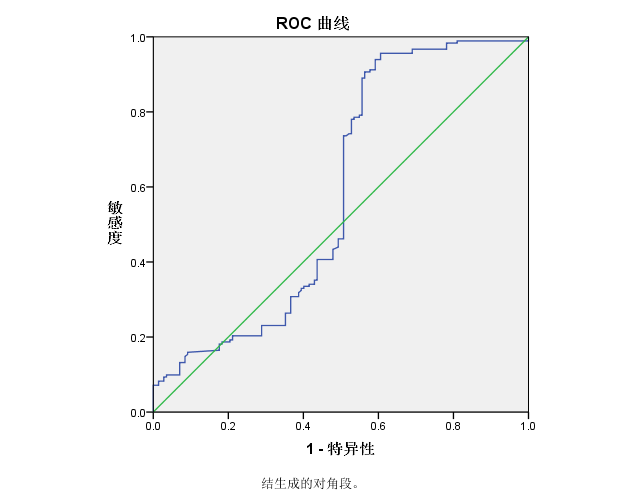


Figue S2 ROC curve of serum irisin levels
